# A rapid preliminary prediction model for intestinal necrosis in acute mesenteric ischemia: a retrospective study

**DOI:** 10.1186/s12876-021-01746-0

**Published:** 2021-04-07

**Authors:** Xinsuo Zhuang, Fumei Chen, Qian Zhou, Yuanrun Zhu, Xiaofeng Yang

**Affiliations:** Emergency and Trauma Center, The International Medical Center, The First Affiliated Hospital, Zhejiang University School of Medicine, 1367 West Wenyi Rd.Zhejiang Province, Hangzhou, 310058 China

**Keywords:** Clinical prediction model, Intestinal necrosis, Acute mesenteric ischemia

## Abstract

**Background:**

Acute mesenteric ischemia (AMI) is a life-threatening condition. However, there is no accurate method to predict intestinal necrosis in AMI patients that may facilitate early surgical intervention. This study thus aimed to explore a simple and accurate model to predict intestinal necrosis in patients with AMI.

**Methods:**

A single-center retrospective study was performed on the data of 132 AMI patients treated between October 2011 and June 2020. The patients were divided into the intestinal necrosis and non-intestinal necrosis groups. The clinical characteristics and laboratory data were analyzed by univariate analysis, and the variables with statistical significance were further analyzed by multivariate logistic regression analysis. The independent predictors of intestinal necrosis were determined and a logistic prediction model was established. Finally, the accuracy, sensitivity, and specificity of the model in predicting intestinal necrosis were evaluated.

**Results:**

Univariate analysis showed that white blood cell (WBC) count, blood urea nitrogen (BUN) level, neutrophil ratio, prothrombin time (PT), and LnD-dimer were associated with intestinal necrosis. According to logistic regression multivariate analysis, WBC count, BUN level and LnD-dimer were independent predictors of intestinal necrosis. These parameters were used to establish a clinical prediction model of intestinal necrosis (CPMIN) as follows: model score = 0.349 × BUN (mmol/L) + 0.109 × WBC × 10^9^ (10^9^/L) + 0.394 × LnD − Dimer (ug/L) − 7.883. The area under the receiver operating characteristics (ROC) curve of the model was 0.889 (95% confidence interval: 0.833–0.944). Model scores greater than − 0.1992 predicted the onset of intestinal necrosis. The accuracy, specificity, and sensitivity of the model were 82.6%, 78.2%, and 88.3%, respectively. The proportion of intestinal necrosis in the high-risk patient group (CPMIN score ≥ − 0.1992) was much greater than that in the low-risk patient group (CPMIN score < − 0.1992; 82.7% vs. 15.0%, *p* < 0.001).

**Conclusion:**

The CPMIN can effectively predict intestinal necrosis and guide early surgical intervention to improve patient prognosis. Patients with AMI who are classified as high-risk should be promptly treated with surgery to avoid the potential complications caused by delayed operation. Patients classified as low-risk group can receive non-surgical treatment. This model may help to lower the morbidity and mortality from AMI. However, this model’s accuracy should be validated by larger sample size studies in the future.

## Introduction

Acute mesenteric ischemia (AMI) is a very serious gastrointestinal tract disorder with high mortality that can result from mesenteric artery embolism, mesenteric artery thrombosis or mesenteric vein thrombosis [1]. An interruption of the blood supply to the mesenteric vasculature can cause necrosis of the small intestine, which can subsequently lead to serious illness and death. The short-term mortality of AMI is high, ranging from 26 to 86% [2–4], and early diagnosis and timely treatment essential to improve the prognosis of AMI [5].

With the continuous improvement of computed tomography (CT) technology–particularly for patients with acute abdominal conditions–AMI can now be diagnosed in the early stages of ischemia [6]. For patients with acute ischemic bowel disease, fasting, blood volume resuscitation, oral and /or intravenous antibiotics, antiplatelets, active anticoagulation therapy and interventional therapy can be initiated if intestinal necrosis has not yet developed. Thus, early intervention can save viable intestine and improve patient prognosis [7–12].

However, it is difficult to quickly and accurately determine whether intestinal necrosis has occurred using current diagnostic methods. Typically, intestinal necrosis is considered only when the patient has apparent peritoneal irritation, bloody ascites on abdominal puncture, and exhibits worsening systemic symptoms [13, 14]. The delay in diagnosing intestinal necrosis often leads to additional intestinal tissue death, which aggravates the patient’s systemic inflammatory response. Patient mortality is therefore closely related to the development of intestinal necrosis [15]. Late surgical treatment also has limitations as extensive intestinal resection leads to short bowel syndrome in surviving patients [16].

Currently, there are few studies that examine early predictors of intestinal necrosis caused by AMI, and no consensus has been reached in this regard. Therefore, to facilitate clinical decision-making for AMI patients, we investigated the clinical and laboratory factors related to small intestine necrosis in AMI patients. Furthermore, we constructed a rapid and simple predictive model to guide treatment for patients at high risk of intestinal necrosis with the goal of reducing patient mortality from this condition.

## Methods

### Patients

A retrospective analysis was performed on the data of AMI patients who visited the Emergency Department of the First Affiliated Hospital of Zhejiang University School of Medicine between October 2011 and June 2020. All patients with acute abdominal complaints who visited the hospital during this timeframe were evaluated. The diagnostic criteria for AMI were symptoms of acute abdominal pain and the presence of superior mesenteric artery or vein embolism confirmed by enhanced CT examination. The exclusion criteria were chronic mesenteric ischemia without acute intestinal injury, tumor-related mesenteric ischemia, acute abdominal or intestinal obstruction secondary to diseases other than AMI, presence of underlying renal function, and patient records missing essential data required for the study.

The patients were divided into the intestinal necrosis and non-intestinal necrosis groups. Intestinal necrosis was diagnosed by surgery in all patients with the disease; diagnosis was confirmed by pathological examination of the excised specimens. All specimens were pathologically examined to confirm full thickness small bowel wall necrosis.

Ethics approval for this study was obtained from the Ethics Committee of the First Affiliated Hospital of Zhejiang University School of Medicine. Precautions were taken to protect the privacy of the research subjects and their information. Informed consent was obtained from all patients.

### Treatment

Based on the time of symptom onset, clinical symptoms, and the results of physical, laboratory, and CT examinations, patients with suspected intestinal necrosis were treated by laparotomy. The specific surgical procedure was determined according to the patient’s condition, with options including intestinal resection, arterial embolectomy, and open-close surgery. Patients without intestinal necrosis were treated medically with anticoagulation therapy, thrombolytic therapy, interventional therapy, or other strategies as appropriate. Interventional therapy includes angiography, balloon dilatation, stent implantation and intravascular thrombolysis. Their condition was closely monitored, and those with worsening conditions were treated surgically.

### Data collection

The following data was collected from the study population: age, gender, cardiovascular disease status, cause(s) of AMI, treatment status, D-dimer level, complete blood count, and measures of hepatic, renal, and coagulation functions. Relevant test and examination results performed within 1 h of patient arrival at the emergency department were considered.

### Statistical analysis

All analysis was performed using SPSS software version 21.0 (SPSS Inc., Chicago, IL, USA). Continuous variables were expressed as mean ± standard deviation (SD) or range, depending on the distribution. Categorical data were expressed as values and percentage. Differences between the two groups were determined by Student’s t-test for normally distributed continuous variables. The Chi-square test or Fisher’s exact test was used in the analysis of qualitative variables. The total WBC, neutrophil, Cr, BUN, PT and lnD-dimer level were significant (*p* < 0.05) through univariate analysis. Those six variables were selected and entered future analysis. To identify the risk factors of intestinal necrosis, a multivariate analysis was performed using a logistic regression model with forward elimination. Results of the multivariate analysis were presented as an odds ratio (OR) (95% confidence interval). WBC count, BUN, and LnD-dimer level were finally entered the preliminary prediction model through multivariate logistic regression analysis. A receiver operating characteristic (ROC) curve was constructed to determine the optimal threshold of continuous factors in predicting recurrence. *p* < 0.05 was considered to be statistically significant.

## Results

### Overview

A total of 132 patients with AMI were included in this study. The clinical and pathologic characteristics of the patients are summarized in Table 1. Of the 132 patients with abdominal pain, 55 patients had intestinal necrosis that was confirmed by surgery and pathological examination. There were 40 female and 92 male patients with a mean age of 62 ± 16.5 (range: 23–93) years. Furthermore, 56 patients had hypertension, and 43 had atrial fibrillation. AMI was caused by arterial embolism in 81 patients and by venous embolism in 51 patients; 72 patients received surgical treatment.

**Table 1:** Characteristics of patients with necrosis and without necrosis

Characteristics	Necrosis group (n = 55)		Non-necrosis group (n = 77)	*p* value	
Age,mean (SD)	64.38 ± 15.82		61.03 ± 16.97	0.251	
Female n (%)	15 (27)		25 (32)	0.522	
Male n (%)	40 (73)		52 (68)		
History of cardiovascular disease n (%)					
Arterial hypertension	23 (42)		33 (43)	0.905	
Atrial fibrillation	16 (30)		27 (35)	0.470	
AMI etiology n (%)				0.586	
Arterial	32 (58)		49 (64)		
Venous	23 (42)		28 (36)		
Therapy n (%)				< 0.001	
Anticoagulation	0 (0)		38 (49)		
Operation	55 (100)		17 (22)		
Intervention	0 (0)		20 (26)		
Thrombolysis	0 (0)		2 (3)		

There were no significant differences in age, gender, history of cardiovascular disease and etiology between AMI patients with TIN and without TIN. The treatment was significant difference between the two groups

### Predictive factors of intestinal necrosis

The results of the univariate analysis of factors associated with intestinal necrosis are shown in Tables 1 and 2. The total white blood cell (WBC) count (*p* < 0.001), neutrophil count (*p* < 0.001), creatinine (*p* = 0.001), blood urea nitrogen (BUN) level (*p* < 0.001), prothrombin time (*p* = 0.026) and LnD-dimer level (*p* < 0.001) were significantly elevated in the intestinal necrosis group compared to the non-necrosis group. There were no differences between the two groups with regards to coagulation function and aspartate aminotransferase level. Multivariable analysis using binary logistic regression showed that the significant independent predictors of intestinal necrosis in patients with AMI were WBC count (OR: 1.115, 95% CI: 1.040–1.196), BUN level (OR: 1.418, 95% CI:1.183–1.699) and LnD-dimer level (OR: 1.483, 95% CI: 1.023–2.149). These results are summarized in Table 3.

**Table 2:** Laboratory examination in patients with acute mesenteric ischemia

Variables (mean ± SD)	Necrosis group (n = 55)	Non-necrosis group (n = 77)	P value
WBC × 10^9^/L	21.68 ± 9.00	12.95 ± 6.44	< 0.001
Hemoglobin, g/L	134.89 ± 9.00	141.18 ± 32.19	0.236
PLT × 10^9^/L	195.91 ± 115.22	213.23 ± 119.36	0.406
Neutrophil × 10^9^/L	19.09 ± 8.13	10.86 ± 6.38	< 0.001
Lymphocyte × 10^9^/L	1.06 ± 0.93	1.18 ± 0.75	0.428
AST, U/L	39.60 ± 43.54	28.67 ± 17.80	0.083
Cr, mmol/L	113.56 ± 83.15	73.04 ± 30.80	0.001
BUN, mmol/L	9.62 ± 4.27	5.48 ± 2.54	< 0.001
PT, S	15.02 ± 6.91	12.86 ± 1.47	0.026
APTT, S	36.14 ± 18.70	31.43 ± 7.68	0.082
lnD-dimer, ug/L	8.70 ± 1.33	7.76 ± 1.31	< 0.001

The total white blood cell (WBC) count (*p* < 0.001), neutrophil count (*p* < 0.001), creatinine (*p* = 0.001), blood urea nitrogen (BUN) level (*p* < 0.001), prothrombin time (*p* = 0.026) and LnD-dimer level (*p* < 0.001) were significantly elevated in the intestinal necrosis group compared to the non-necrosis group. There were no differences between the two groups with regards to coagulation function and aspartate aminotransferase level

WBC, white blood cell; PLT, the platelet count; AST, aspartate aminotransferase; Cr, creatinine; BUN, blood urea nitrogen; PT, prothrombin time; APTT, activated partial thromboplastin time

**Table 3:** Multivariable analysis of factors associated with necrosis

Variable	*p* value	B	SE	Wald Chi-squar	OR	95% CI for OR
WBC	0.002	0.109	0.036	9.332	1.115	1.040–1.196
BUN	0.000	0.349	0.092	14.326	1.418	1.183–1.699
lnD-dimer	0.038	0.394	0.189	4.326	1.483	1.023–2.14

Multivariable analysis using binary logistic regression showed that the significant independent predictors of intestinal necrosis in patients with AMI were WBC count (OR: 1.115, 95% CI 1.040–1.196), BUN level (OR: 1.418, 95% CI 1.183–1.699) and LnD-dimer level (OR: 1.483, 95% CI 1.023–2.149)

WBC, white blood cell; BUN, blood urea nitrogen

### A clinical prediction model for intestinal necrosis (CPMIN)

**Fig. 1:**
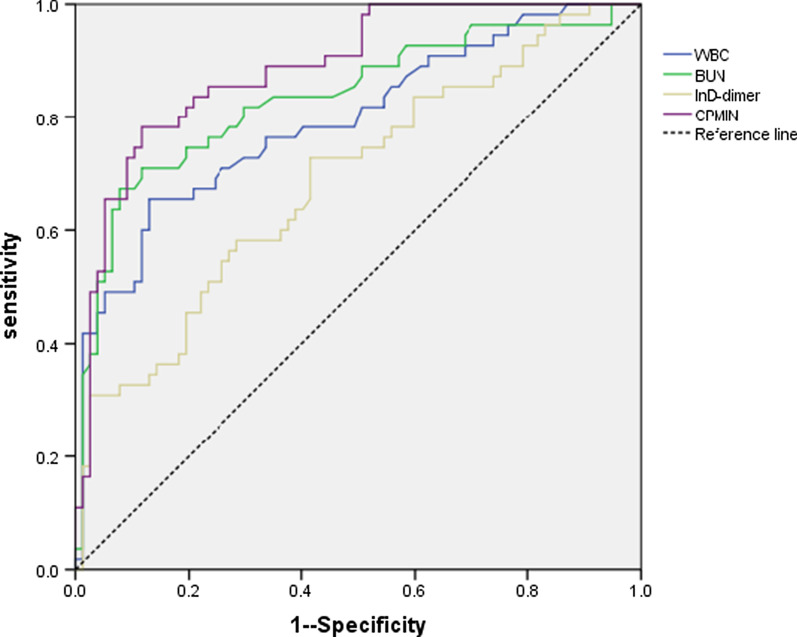
AUROC of the PMIN for predicting the incidence of intestinal necrosis. The area under the curve of the model was 0.889 (95% CI 0.833–0.944), with a standard error of 0.029

WBC count, BUN, and LnD-dimer level were significantly correlated with intestinal necrosis as determined by multivariate logistic regression analysis. Based on this analysis, the clinical prediction model for intestinal necrosis (CPMIN) was established as follows: model score = 0.349 × BUN (mmol/L) + 0.109 × WBC × 10^9^ (10^9^/L) + 0.394 × LnD − Dimer (ug/L) − 7.883. The area under the receiver operating characteristics (ROC) curve of the model was 0.889 (95% CI: 0.833–0.944); the standard error of the model was 0.029 (Fig. 1). Using the ROC curve, the threshold value of the CPMIN for predicting the onset of intestinal necrosis was − 0.1992. Based on this value, the accuracy, sensitivity and specificity of the model were 84.1%, 78.2%, and 88.3%, respectively.

**Fig. 2:**
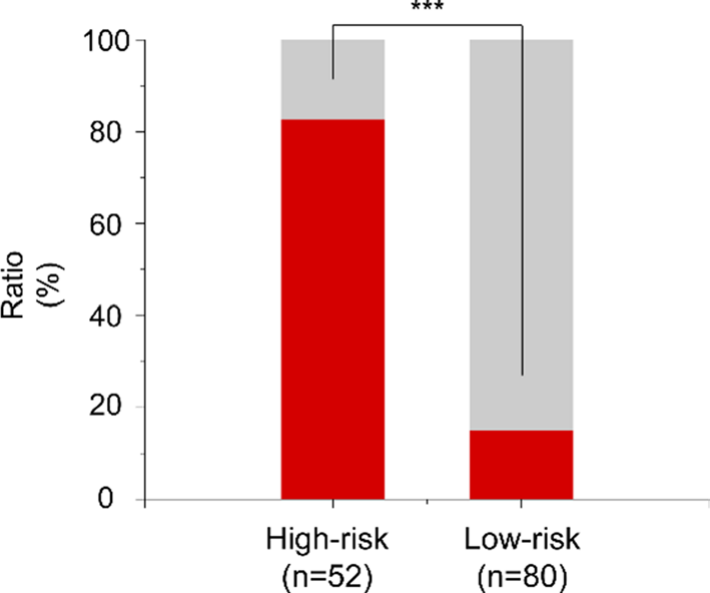
The proportion of intestinal necrosis in the different groups. The proportion of intestinal necrosis in the high-risk group was much higher than that in the low-risk group (82.7% vs.15.0%, *p* < 0.001)

**Fig. 3:**
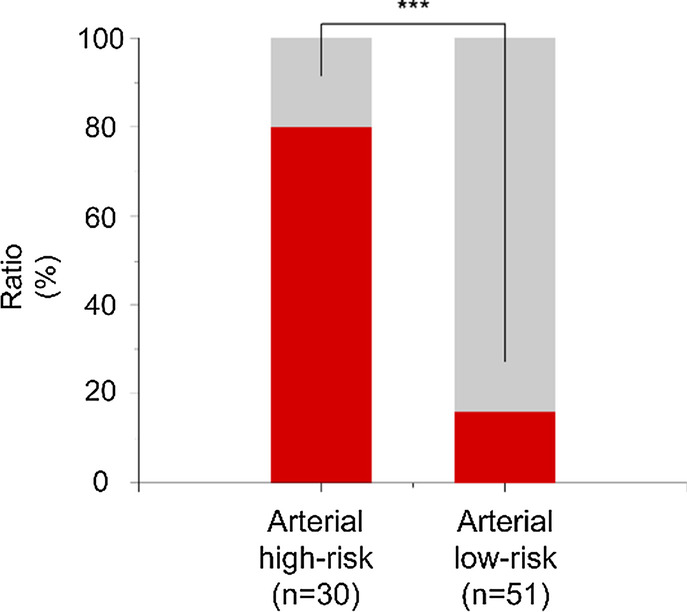
The proportion of intestinal necrosis in the arterial group. The proportion of the intestinal necrosis in arterial high-risk group was significantly higher than that in low-risk group (80.0% vs.15.7%, *p* < 0.001)

**Fig. 4:**
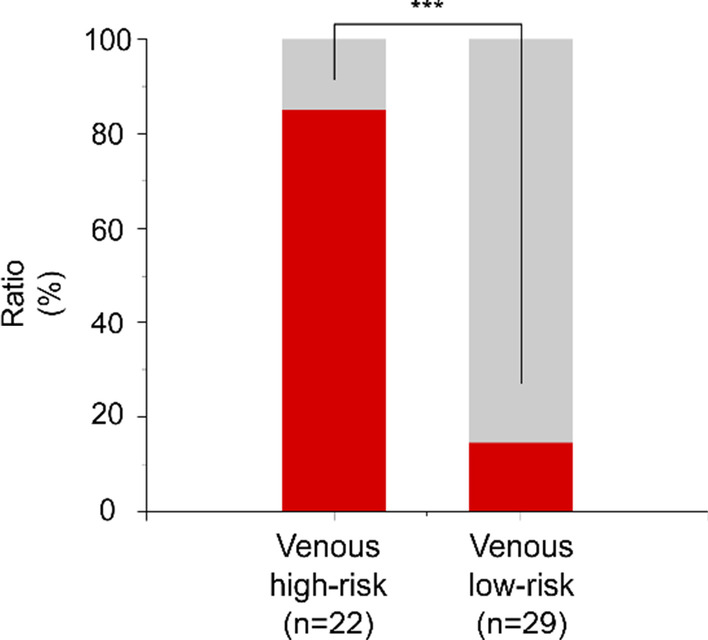
The proportion of the intestinal necrosis in venous group. The proportion of the intestinal necrosis in venous high-risk group was significantly higher than that in low-risk group (86.4% vs.13.7%, *p* < 0.001)

Patients were divided into two groups based on their CPMIN score: a high-risk group (CPMIN scores ≥ − 0.1992, n = 52) and low-risk group (CPMIN scores < − 0.1992, n = 80). The proportion of patients with intestinal necrosis in the high-risk group was significantly greater than that in the low-risk group (82.7% vs. 15.0%, *p* < 0.001) (Fig. 2). Additionally, according to the etiology of AMI and the CPMIN score, patients were subdivided into four groups: the arterial high-risk (CPMIN score ≥ − 0.1992, n = 30), arterial low-risk (CPMIN scores < − 0.1992, n = 51), venous high-risk (CPMIN scores ≥ − 0.1992, n = 22), and venous low-risk (CPMIN scores < − 0.1992, n = 29) groups. The proportion of patients with intestinal necrosis in the arterial high-risk group was significantly higher than that in the arterial low-risk group (80.0% vs. 15.7%, *p* < 0.001; Fig. 3); the proportion of patients with intestinal necrosis in the venous high-risk group was also significantly higher than that in the venous low-risk group (86.4% vs. 13.7%, *p* < 0.001; Fig. 4).

## Discussion

Early diagnosis of AMI is very important for the prognosis of patients. Patients with suspected AMI should be examined with enhanced CT as soon as possible [17]. A management strategy including oral antibiotics in addition to early revascularization might reduce or prevent progression of AMI towards irreversible transmural intestinal necrosis (ITIN) [12]. The establishment of Intestinal Stroke Center can provide multimodal and multidisciplinary management, the modern treatment of mesenteric ischemia in Intestinal Stroke Centers has allowed rates of resection-free survival in nearly two-thirds of patients [10, 11].

Prompt and appropriate treatment is required when intestinal necrosis occurs in patients with AMI. Surgical delays often lead to death due to disease complications such as acute intestinal failure, sepsis, multiple organ failure [18–20], peritonitis, and short bowel syndrome in advanced or unresected intestinal necrosis [5, 21]. The extent of the small intestine resection is independently associated with initial mortality [22–24]. Thus, intestinal necrosis should be identified and tissue should be removed before clinical symptoms of peritonitis and organ failure appear in order to reduce morbidity and mortality and to improve intestinal function. In addition, accurate preoperative screening can avoid unnecessary laparotomy and resection in some patients with non-transmural intestinal necrosis [25, 26]. This research may help identify the criteria for emergency surgery based on clinical presentation.

We identified BUN level, LnD-dimer level and WBC count as the parameters predictive of intestinal necrosis in AMI patients. Urea nitrogen is the primary end-product of human protein metabolism and is predominantly excreted by the kidney. When the glomerular filtration rate drops below 50% of the standard value, BUN rises rapidly. Accordingly, elevated BUN is one of the leading indicators for renal function testing. After ischemic necrosis of the small intestine, intestinal cells become hypoxic. As a result, anaerobic glycolysis is enhanced and lactic acid production increases, leading to hypoxia-associated changes in renal cells that subsequently cause inflammation. As necrosis of the small intestine progresses, blood volume, cardiac output, and renal blood flow decrease leading to increased levels of renally toxic substances in the blood; in turn, this process affects renal function and leads to increased BUN levels [27]. Wei [28] et al. found that the BUN levels significantly increase when necrosis of the small intestine occurs, including in patients with AMI. However, there are currently few studies on this association; thus, further investigation of the relationship between BUN level and ischemic necrosis of the small intestine is required.

D-dimer is the end-product of fibrin degradation and is often used for diagnosis- by-exclusion of venous thrombosis [29, 30]. There are few studies on the dynamic level of D-dimer in AMI. Intestinal necrosis causes microcirculation, blood coagulation, and blood circulation disturbances in the intestine, while tissue ischemia and hypoxia can cause coagulation disorders and microthrombosis. Therefore, coagulation function markers such as D-dimer and fibrinogen can indirectly reflect the blood circulation to the diseased abdominal organs [31–33]. We found that the D-dimer levels were significantly different between patients in the intestinal necrosis group and the non-intestinal necrosis group. This also indicates that an increase in the level of plasma D-dimer suggests intestinal necrosis.

When a patient develops ischemic necrosis of the intestines, the permeability of the intestinal wall decreases, and bacteria in the intestine can migrate into the abdominal cavity. This causes abdominal cavity infection, leading to an increase in WBC count [34]. In the context of AMI, elevated WBC count therefore indicates that intestinal strangulation or necrosis should be considered [35, 36]. Kassahun [37] found that an increase in WBC counts can be used as an indicator of ischemic necrosis of the small intestine through analysis of clinical manifestations, laboratory examinations, and imaging of 60 cases of ischemic necrosis of the small intestine, which can support the decision to perform surgery for intestinal necrosis. Consistent with this result, we identified a statistically significant difference in WBC counts between patients in the intestinal necrosis group and the non-intestinal necrosis group.

**Fig. 5:**
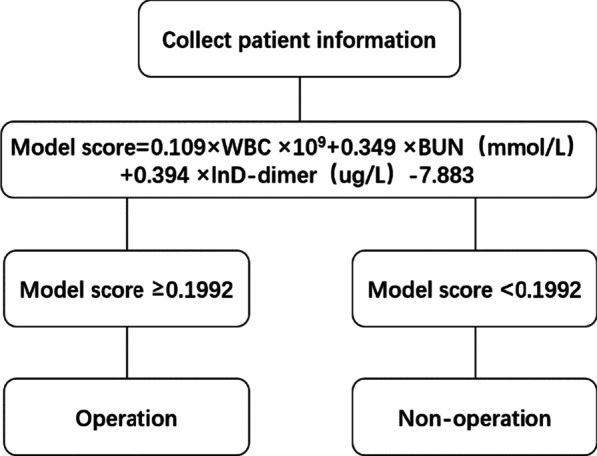
Example of management proposal using CPMIN assessment in treatment decision in acute mesenteric ischemia. The CPMIN guides clinical decision-making. If the model score ≥ − 0.1992, surgery is recommended. If the model score < − 0.1992, conservative treatment can be taken

The predictive model of intestinal necrosis based on serum BUN and plasma D-dimer levels as well as WBC counts offers a useful tool to guide clinical decision making for AMI patients. Most previous studies on this topic have focused on inflammatory or imaging indicators to predict the need for bowel resection, but biochemical indicators have rarely been included. Our model integrates inflammatory, thrombus-related, and biochemical indicators. Based on the accuracy of our model, the CPMIN can effectively predict intestinal necrosis and guide precise early intervention. Patients with high-risk AMI (CPMIN score ≥ − 0.1992) should be promptly treated with surgery to avoid the various potential complications of delayed operation, whereas Patients in the low-risk AMI group (CPMIN score < − 0.1992) can receive non-surgical treatment (Fig. 5). Thus, using the CPMIN may help to reduce the overall mortality of intestinal necrosis.

This study was somewhat limited by its small sample size, which may have resulted in some bias. This model’s accuracy should be validated by larger sample size studies in the future. In the subsequent research, we will expand the sample size and/or conduct a multi-center study to further validate the CPMIN. We note that numerous studies identified lactate levels as major and independent biomarker of intestinal necrosis [26, 35, 38, 39]. In this study, some of the patients were in mild condition, and the collected data were within 1 h after the patients arrived at the emergency department. Some patients did not have lactate test. Therefore, lactate was not included in this study. We will include the study of lactate in the follow-up study. Furthermore, imaging data were not included in this study, but will be examined in follow-up studies. Professional radiologists will be invited to guide data collection and analysis. Finally, it was found in this study that some patients in the low-risk AMI group still had intestinal necrosis; further research is needed to reliably identify these patients. Nonetheless, the CPMIN presented here based on BUN level, WBC count, and D-dimer level is a simple and effective predictive model of intestinal necrosis in the context of AMI.

## Data Availability

The datasets generated and/or analyzed in the present study are available from the corresponding author on reasonable request.
